# A Hierarchy of Patient-Reported Outcomes for Meta-Analysis of Knee Osteoarthritis Trials: Empirical Evidence from a Survey of High Impact Journals

**DOI:** 10.1155/2012/136245

**Published:** 2012-06-26

**Authors:** Carsten Juhl, Hans Lund, Ewa M. Roos, Weiya Zhang, Robin Christensen

**Affiliations:** ^1^Research Unit for Musculoskeletal Function and Physiotherapy, Institute of Sports Science and Clinical Biomechanics, University of Southern Denmark, 5230 Odense M, Denmark; ^2^Department of Physiotherapy, Copenhagen University Hospital, Gentofte, 2900 Hellerup, Denmark; ^3^Academic Rheumatology, University of Nottingham, Nottingham NG5 1PB, UK; ^4^Musculoskeletal Statistics Unit, The Parker Institute, Copenhagen University Hospital, Frederiksberg, 2000 Frederiksberg, Denmark

## Abstract

*Objectives*. To develop a prioritised list based on responsiveness for extracting patient-reported outcomes (PROs) measuring pain and disability for performing meta-analyses in knee osteoarthritis (OA). *Methods*. A systematic search was conducted in 20 highest impact factor general and rheumatology journals chosen a priori. Eligible studies were randomised controlled trials, using two or more PROs measuring pain and/or disability. *Results*. A literature search identified 402 publications and 38 trials were included, resulting in 54 randomised comparisons. Thirty-five trials had sufficient data on pain and 15 trials on disability. The WOMAC “pain” and “function” subscales were the most responsive composite scores. The following list was developed. Pain: (1) WOMAC “pain” subscale, (2) pain during activity (VAS), (3) pain during walking (VAS), (4) general knee pain (VAS), (5) pain at rest (VAS), (6) other composite pain scales, and (7) other single item measures. Disability: (1) WOMAC “function” subscale, (2) SF-36 “physical function” subscale, (3) SF-36 (Physical composite score), and (4) Other composite disability scores. *Conclusions*. As choosing the PRO most favourable for the intervention from individual trials can lead to biased estimates, using a prioritised list as developed in this study is recommended to reduce risk of biased selection of PROs in meta-analyses.

## 1. Introduction

Measures for patient-reported outcomes (PROs) are used in most osteoarthritis (OA) trials. Extracting and combining these data is an essential part of any meta-analysis of such trials. At the 3rd Outcome Measures in Rheumatology (OMERACT) Conference in 1996, consensus was reached on three domains—pain, disability, and patient global assessment—as measures that should be reported in future OA trials [1]. This recommendation only specified which constructs to measure, but not which specific instruments to include [2]. In knee OA, pain is typically measured by either the Western Ontario and McMaster Universities Osteoarthritis Index (WOMAC, “pain” subscale) or by different pain scores using a visual analogue scale (VAS). Patient-reported disability is often measured by WOMAC “function” subscale or Short Form 36 (SF-36 “physical functioning” (PF) subscale), but a variety of different PRO measures have been used [3]. Often several PROs are used in trials for estimating change in pain and disability. Simply extracting data on the primary outcome may not be an option in a meta-analysis, as this is often not reported and may not be available for both pain and disability. If the choice of PROs is based on which outcome measure reaches statistical significance, the corresponding meta-analytic estimates are likely to be biased [4, 5].

Ideally, the most responsive outcome measure is the best choice for extraction and inclusion in a meta-analysis, provided that it is a valid outcome measure and included in the majority of the trials. Whether the outcome measure is truly valid (i.e., it measures the intended change) is a separate consideration based on face, content, construct, and criterion validity [6]. Good validity is a prerequisite for high responsiveness [7]. Responsiveness is quantified as the standardised mean difference (SMD), which is calculated as the difference in the mean change between the intervention and control groups divided by the pooled standard deviation (combining the different groups in any particular trial). The PRO with the largest SMD is considered to be the most responsive [8].

The choice of the most responsive among the potential outcome measures is consistent with the OMERACT filter of truth, discrimination, and feasibility [9]. Combining instruments with different responsiveness can increase heterogeneity in a meta-analysis [10]. Alternatively, various outcomes can be transformed to the most frequently used outcome, using the transformation coefficients from regression analyses [7, 10].

In meta-analyses of OA, it is recommended that a hierarchy of PROs should be determined prior to data extraction [11]. In 2006, Jüni et al. suggested a prioritised list for extracting data on pain and disability in patients with knee OA, but the methodology behind the list was not reported [11]. Likewise in a meta-analysis of aquatic exercise for OA, a nonsystematic approach was used to develop an operational hierarchy for OA meta-analyses [12].

### 1.1. Objectives

The aim of this study was to develop a prioritised list for extracting PROs on pain and disability for meta-analyses, based on an investigation of the responsiveness of PRO measures in knee OA, and restricted to trials with an anticipated low risk of selective outcome reporting.

## 2. Methods

### 2.1. Eligibility Criteria

Trials were considered eligible in the current study if they were designed as randomised controlled trials (RCTs) or quasi-randomised trials investigating any type of intervention for patients with knee OA [13]. Trials had to include at least one group with a specified intervention and a control group. In accordance with international consensus regarding the core set of outcome measures for phase III clinical trials in OA [9], the eligible RCTs had to include assessment of at least self-reported pain and/or self-reported disability. Only trials measuring one or both of these constructs with at least two different outcome measures were eligible.

### 2.2. Information Sources

A systematic literature search was conducted in MEDLINE in December 2009 by searching PubMed in order to identify RCTs of interventions aimed at reducing pain and disability in patients with knee OA. In order to identify RCTs with a low risk of selective outcome reporting, the search was restricted to RCTs published later than the year of 2002. The revised CONSORT Statement was published in 2001, and the reporting of RCTs was expected to improve from 2002 [14].

### 2.3. Literature Search

The search terms for identifying knee OA were “osteoarthritis, knee” [MeSH] OR (“osteoarthritis” [TIAB] AND “knee” [TIAB]). The search was restricted to the ten highest impact factor general and internal medicine journals (previous 5 years) as well as the ten highest impact factor rheumatology journals (previous 5 years) (determined by Journal Citation Reports, http://isiwebofknowledge.com. 6 November 2009): The New England Journal of The Medicine; Journal of the American Medical Association; The Lancet; Annals of Internal Medicine; British Medical Journal; PLoS Medicine; Annual Review of Medicine; Archives of Internal Medicine; Canadian Medical Association Journal; Medicine, Arthritis and Rheumatism; Annals of Rheumatic Diseases; Nature Clinical Practice Rheumatology; Arthritis Research & Therapy; Seminars in Arthritis and Rheumatism; Osteoarthritis and Cartilage, Rheumatology (Oxford); Current Opinion in Rheumatology; Journal of Rheumatology; and Best Practice and Research in Clinical Rheumatology. Targeting journals with a high impact factor has previously been suggested as a good strategy for identifying journal articles with high methodological quality [15].

### 2.4. Study Selection

Two members of the study team (C. Juhl, H. Lund) independently scrutinised titles and abstracts of all identified publications. The full text of any article was obtained if it was judged eligible by at least one of the reviewers. The two reviewers then evaluated eligibility based on the full text of all the retrieved papers, and consensus on inclusion was reached by discussion.

### 2.5. Data Collections Process and Data Items

Study identification (author, year) and outcome measures were extracted using a customised data extraction form. For each outcome measure, the number of participants in the intervention and the control groups and the mean change and standard deviation (SD) were extracted in order to calculate the SMD. When SD was not available in an explicit format, it was estimated from the standard error (SE), confidence interval, the *P* value, the interquartile range, or other methods as recommended by the Cochrane Collaboration [16].

### 2.6. Risk of Bias in Individual Studies

Selective outcome reporting has been defined as choosing a subset of the original outcomes on the basis of the results [1, 2]. Two members of the study team (C. Juhl, H. Lund) assessed the risk of selective outcome reporting, indexed according to whether the trials had been classified as “adequate,” “unclear,” or “inadequate” in accordance with the Cochrane Handbook for Systematic Reviews of Interventions 5.1.0 [16]. Selective outcome reporting was deemed as follows.

- Adequate, if a protocol (published or from ClinicalTrials gov or other databases) was available and all PROs were sufficiently reported for extracting data for estimating SMD.
- Unclear, if a protocol was not available (published or from ClinicalTrials gov or other databases).
- Inadequate, if some or all PROs were insufficiently reported for extracting data for estimating SMD (evaluated by checking the protocol from earlier publications, trial registers, or described in the published trial).

### 2.7. Summary Measures

The effect size was calculated as the SMD to allow comparison of the various PROs. The SMD was estimated as the difference in mean change between the intervention and control groups divided by the pooled SD. The pooled SD was estimated from SD_pooled_^2^ = (SD_*I*_^2^ × [*N*_*I*_ − 1] + SD_*C*_^2^ × [*N*_*C*_ − 1])/(*N*_*I*_ + *N*_*C*_ − 2), where *N*_*I*_ and SD_*I*_ represent the number of patients and the SD in the intervention group, respectively. The SMD was used to represent the responsiveness; the higher the SMD, the more responsive the measure. This approach is only valid to rank PROs within a particular study, as different studies obviously measure different therapeutic interventions. Data from an intention to treat (ITT) analysis was preferred for calculating the SMD. When several intervention groups were compared with a control group, the number of control patients was divided equally into the appropriate number of groups when estimating the SMD.

### 2.8. Synthesis of Results

The responsiveness estimated as SMD of the PROs in each of the included trials (or subgroups when more interventions were compared with the control) was ranked according to responsiveness for pain and disability separately. The PRO used to measure the effect of an intervention in any individual trial with the highest responsiveness was ranked 1, the second most responsive was ranked 2, and so on. The mean rank was then used to estimate the responsiveness across the trials, and a low mean rank (close to 1) indicated that this PRO was often the most responsive PRO used. The PROs used in at least 5 trials were then listed according to the lowest mean rank of the SMD. However, composite item scales with established validity would rate higher than single item scales.

### 2.9. Risk of Bias across Studies

A sensitivity analysis was performed evaluating the impact of different systematic approaches to data extraction of PROs in a meta-analysis. The pooled mean across trials using the developed list from the current study was compared with lists based on (1) the most favourable outcome from each of the individual trials, (2) the most frequently used PROs, and (3) the most responsive of the PROs. Inconsistency in means between trials was evaluated using the *I* ^2^ index [17]. A random effect model was used for pooling the trials. All analyses were performed at the study level using the meta-analysis software “Comprehensive Meta Analysis” Version 2, Biostat. Inc., and were based on published data only.

### 2.10. Additional Analysis

In order to assess the robustness of the developed list, subgroup analyses stratifying the available trials according to risk of selective outcome bias were applied, and a list based on trials with no risk of selective outcome bias was compared with the list based on all included trials. Stratified analyses were performed based on whether the included trials were published in the general/internal medicine journals or in the rheumatology journals. Secondly, sensitivity analyses were performed stratifying trials according to the intervention; injection in the knee joint, oral medication and other interventions (tai chi, lateral wedge shoes, etc.). Outcomes used at least 5 times were ranked based on the mean rank and the lists from these subgroups were compared to the list based of all included trials.

## 3. Results

### 3.1. Study Selection

**Figure 1:**
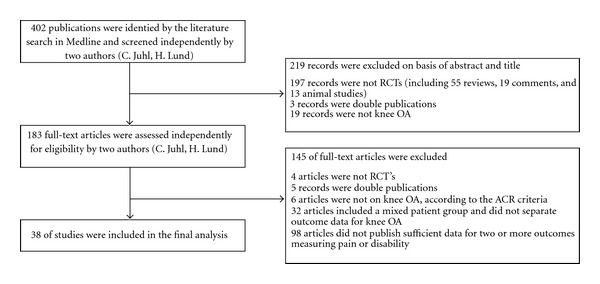
Flowchart: identification of studies.

Through the search strategy, 402 publications were identified, as presented in the flowchart (Figure 1). Titles and abstracts of the publications were checked independently by two reviewers (C. Juhl, H. Lund). One hundred and eighty three trials were identified as potentially eligible by at least one of the reviewers and subsequently examined independently in full text by two reviewers (C. Juhl, H. Lund). Consensus was reached by discussion and resulted in 38 trials that fulfilled the eligibility criteria and were included in the analysis [18–55] (Table 1).

### 3.2. Study Characteristics

Pain was evaluated with more than one PRO in 35 trials [18–21, 23–42, 45–55], and disability was evaluated in 15 trials [18, 19, 21, 22, 25, 26, 29, 31, 38, 40, 43, 44, 46, 53, 54]. More than one intervention group was compared in 14 trials measuring pain [19, 24, 25, 27–29, 34–36, 41, 42, 48–50, 55] and in 4 trials measuring disability [22, 25, 29, 40]. Different specific questions were asked when VAS scores were used for measuring pain. The VAS scores were classified as pain during activity (e.g., stepping, daily activities, etc.), pain during walking, pain at rest, pain at night, and general knee pain. WOMAC was used both in a VAS version using a 100 mm scale and in a Likert version using a 1–5 scale, and these were treated as different outcomes in the initial analysis. Pain during activity and pain during walking were measured by either a VAS score or a numeric rating scale (NRS) score, and these PROs were analysed separately. Then eighteen different PROs were used for measuring pain and seven PROs for measuring disability in the included trials.

### 3.3. Risk of Bias within Studies

Only 12 out of the 38 included trials were registered in ClinicalTrial.gov. Six of these trials were classified as “adequate” and six as “inadequate”. Two trials reported PROs not declared in the protocol, one did not report all PROs from the published protocol, one did not report all time points, and two protocols did not report PROs at all in the published protocol (Table 1).

### 3.4. Results of Individual Studies

The most frequently used PROs for measuring pain were the WOMAC “pain” subscale (in either the Likert scale or VAS format) used in 27 of the included trials and for disability the most frequently used PRO was the WOMAC “function” subscale (in either the Likert scale or VAS format) used in all 15 trials (Table 2).

The most responsive PRO measure for pain was “pain during activity” using a VAS with a mean rank of 1.4. It was used when comparing the effect of an intervention in 12 trials (comparing 18 interventions with controls), and it was the most responsive outcome in 14 of these 18 substudies. The most responsive composite score for pain was the WOMAC “pain” subscale using a Likert scale with a mean rank of 1.8. It was the most responsive outcome in 7 out of 20 substudies and the most responsive composite score in 19 out of 20 substudies. The most responsive PRO on disability was WOMAC “function” subscale using a 100 mm scale with a mean rank of 1.4. It was the most responsive outcome in 7 out of 13 substudies.

### 3.5. Synthesis of Results

According to the Food and Drug Administration (FDA), a single item may be reasonable for concepts such as pain severity, if it is a reliable and valid measure, but not for general concepts such as disability [56]. Different single item VAS scores were actually measuring different items, as they were based on different questions. A list based on the responsiveness of the PROs was constructed, placing the most responsive of the PROs as first choice for extracting data for meta-analyses, and PROs based on a single item being downgraded as they were derived from different instruments, whose validity and reliability had not been established. The final prioritised list for preferred PROs to extract when performing meta-analyses is presented in Table 3.

### 3.6. Risk of Bias across Studies

**Figure 2:**
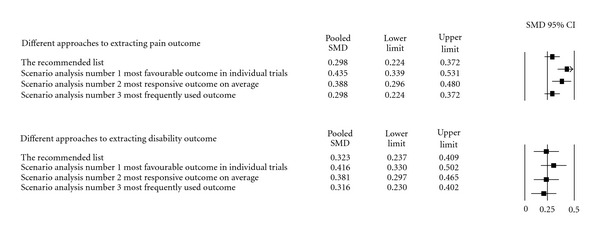
Impact on different approaches to extracting data for meta-analysis.

The likelihood of overestimating the pooled effect in meta-analyses by choosing the most favourable PROs for the intervention in each trial is illustrated in Figure 2. Compared with the developed list, consistently choosing the most favourable PROs overestimated the effect size for pain with SMD = 0.14 (95% CI 0.02, 0.26) and for disability with SMD = 0.09 (95% CI –0.03, 0.21). The differences between (1) the developed list, (2) the most frequently used PROs, and (3) the most responsive of the PROs were smaller. The list developed in this study seems to be a conservative but robust approach for extracting PROs when planning a meta-analysis.

### 3.7. Additional Analysis

The subgroup analysis stratifying the available trials according to risk of selective outcome bias was applied, and a list based on trials with a low risk of selective outcome reporting was developed (i.e., trials with a published protocol and evaluated as “adequate” in the risk of bias assessment). All 6 trials with a low risk of selective outcome reporting had measured pain, but only 3 had measured disability. Even though some outcomes were missing in the list based on these 6 trials with low risk of selective outcome reporting bias compared with the list based on all the included 38 trials, the differences between the two lists were very small (data not shown).

When stratifying the included trials according to the interventions; injection in the knee joint, oral medication and other interventions (tai chi, lateral wedge shoes etc.) no differences in the responsiveness of the most frequently used outcomes (a least 5 times) were found between the “injection subgroup”, the “other interventions subgroup” and all the included trials. In the “oral medication subgroup” the five most responsive outcomes (used a least 5 times) were “Pain walking”, “Pain global”, WOMAC (100 mm scale), “Pain at rest” and WOMAC (likert scale). When compared this to the list of outcomes from all the included trials “Pain activity” and SF-36 is missing as they were not used as frequently in the “oral medication subgroup” and the rank of the last three later were WOMAC (likert scale), WOMAC (100 mm scale) and “Pain at rest” but the responsiveness for these three outcomes was more or less the same. No differences in sensitivity analysis based on intervention were seen in the disability list. Other sensitivity analyses based on whether the included trials were published in the general/internal medicine journals or in the rheumatology journals showed no differences between lists based on mean rank in these subgroups and the list of all trials neither for the analysis of disability nor for pain.

## 4. Discussion

### 4.1. Summary of Evidence

Biased selection of PROs in meta-analyses (e.g., choosing the most favourable PROs for the intervention from individual trials) can overestimate the effect compared with a systematic approach. As anticipated, choosing the “most favourable” outcome from each individual trial was more positive than systematic approaches based on either (1) how frequently the PROs were used, (2) the average responsiveness of the PROs, or (3) the developed list. Using a prioritised list as developed in this study is recommended to reduce the risk of biased selection of PROs in meta-analyses. When comparing the list developed in this study with the hierarchy for extracting pain measurement scales published by Jüni et al. [11], the main differences are that Jüni et al. included global health outcome (as the total WOMAC score, patient's global health and physician's global health), contrasting with the developed list in this study, which only included specific pain measurement scales. The list used by Bartels et al. [12] was based on consensus among the authors and reports the WOMAC subscales as first choice in both pain and disability, similar to the list developed in this study.

### 4.2. Limitations

This study has some limitations. First of all, even though 38 trials were included, and 35 trials had sufficient data on pain, only 15 trials had sufficient data on disability. The literature search was performed in the ten highest impact factor general and internal medicine journals as well as the ten highest impact factor rheumatology journals in order to identify trials with a low risk of selective reporting bias. Some of the highly ranked journals were primarily review journals. Even though these journals only published a few randomised controlled trials, some of the eligible trials were published in one of these journals. Only six trials were classified as “adequate” for having a low risk of selective reporting bias, and some frequently used PROs were missing in the list based on these trials. However, as the differences between the list based on these trials with a low risk of selective outcome reporting bias and the list based on all the included 38 trials were very small, the developed list seems to be trustworthy. Furthermore, stratified analyses comparing lists based on the subgroups of trials and the list from all included trials showed only small discrepancies for the subgroup of “oral medication” using pain as outcome. No differences were seen in other subgroup analyses.

As the subgroup analyses based on whether the trials were published in the general/internal medicine journals or in the rheumatology journals showed no differences between the list from the subgroup and the list from all included trials, it seems unlikely that including more trials will change the list of the most frequently used outcome. So, even though including more journals in the literature search could be preferable, based on these subgroup analyses, it is unlikely that including more trials would change the list developed in this study.

Secondly, combining the SMDs across different populations and interventions can cause heterogeneity, as characteristics of the patient populations (age, sex, BMI, etc.) and interventions have an impact on the effect size. A large SMD can firstly be due to a large difference in mean change between the intervention group and control group, or secondly, to a small standard deviation due to small variability in the included patient group. Combining SMDs across trials could then cause bias if trials with a homogeneous patient group (small SD) were combined with trials with a heterogeneous patient group (large SD). These differences are reduced when using the rank of the SMD instead, when comparing across trials. In developing the prioritised list for extracting PROs in this study, the estimated SMDs were then only used for ranking the PROs in each trial. When the variability in a trial was small due to a homogeneous patient group, the variability in all PROs in the trial was then expected to be equally small with the rank of the SMD still being an acceptable measure of the relationship of the responsiveness between the PROs in individual trials.

Thirdly, as a large number of different PROs were used and most of the included trials only compared two PROs on either pain or disability, it was not possible to make direct comparisons of the responsiveness of PROs. Therefore, the indirect method of ranking the PROs in the individual trials according to responsiveness was used. This method of ranking the PROs reduces the impact of the differences in populations and interventions between the included trials, as it only used the SMD for ranking the responsiveness of the PROs. As the mean rank of the PROs could change if more trials were included, especially the PROs only used in a few trials, the systematically developed list in this study was based on the most frequently used PROs (used in at least 5 trials).

### 4.3. Strengths

A strength of this study was that a comprehensive systematic literature search in high-quality journals was performed, and a systematic approach was used for developing the prioritised list. Furthermore, the impact of using the developed list for data extraction in meta-analyses was analysed. Finally, the prioritised list developed in this study was compared with a list based on the six trials with a low risk of selective outcome reporting bias, and the differences were small.

To summarise, the WOMAC subscales for both pain and disability should be the first choice for extracting PROs in meta-analyses for patients with knee OA. Different single item VAS scores were actually measuring different items (e.g., VAS during activity covers “pain during daily activity,” “pain following stepping activities,” or “pain during worst activity”), so, even though different single item VAS scores for measuring pain were more responsive than the WOMAC, WOMAC is preferred for meta-analysis.

The Knee Injury and Osteoarthritis Outcome Score (KOOS) “ADL” subscale is equivalent to the WOMAC “function” subscale, and the WOMAC “pain” score is contained in the KOOS “pain” score. As the corresponding KOOS subscales and WOMAC subscales have shown equal responsiveness [57], the KOOS subscales could be included in the developed list. Based on these preliminary results and considerations, we recommend the list presented in Table 3, when extracting PROs on pain and disability for meta-analysis.

## 5. Conclusions

As choosing the most favorable patient-reported outcomes (PROs) from individual trials can overestimate the effect compared with a systematic approach, using a prioritised list as presented in this study is recommended to reduce reviewers' likelihood of biased selection of PROs in meta-analyses.

The impact of the prioritised list should be tested in published meta-analyses investigating the effect of interventions on pain and disability for patients with knee osteoarthritis. When a larger number of trials are registered and classified as “adequate” for having a low risk of selective reporting outcome bias, this study should be repeated in order to check the prioritised list based on the responsiveness of PROs.

## Figures and Tables

**Table 1:** Characteristics of included studies.

Author	Journal	Comparison	Number of randomised patients	Construct	Selective outcome reporting	Outcome	Number of patients	Standard mean differences (SMD) measured as Cohens' d (SE)	Rank of responsiveness measured as SMD
Altman et al. (Flexx) 2009	Semin Arthritis Rheum.	Sodium hyaluronate versus saline	588	Pain	Unclear	WOMAC	291/295	0.143 (0.083)	2
						VAS (walking)	285/295	0.259 (0.083)	1
						WOMAC	291/295	0.194 (0.083)	1
				Disability		SF-36 (PCS)	291/295	0.191 (0.083)	2
						SF-36 (PF)	291/295	0.170 (0.083)	3
Baltzer et al. 2009	Osteoarthritis and Cartilage	Autologous conditioned serum (ACS) versus saline	241	Pain	Unclear	WOMAC	134/107	0.703 (0.166)	2
						VAS (activity)	134/107	0.937 (0.169)	1
				Disability		WOMAC	134/107	0.699 (0.166)	1
						SF-8 (PCS)	134/107	0.682 (0.166)	2
		Hyaluronan versus saline	242	Pain	Unclear	WOMAC	135/107	0.050 (0.162)	1
						VAS (activity)	135/107	0.035 (0.162)	2
				Disability		WOMAC	135/107	0.078 (0.162)	2
						SF-8 (PCS)	135/107	0.146 (0.162)	1
Barthel et al. 2009	Semin Arthritis Rheum.	Diclofenac versus vehicle	492	Pain	Unclear	WOMAC	254/238	0.234 (0.091)	2
						VAS (activity)	254/238	0.281 (0.091)	1
Bennell et al. 2005	Ann. Rheum. Dis.	Multimodal physiotherapy programme versus placebo	140	Pain	Unclear	WOMAC	73/67	0.122 (0.169)	3
						VAS (activity)	73/67	0.223 (0.170)	1
						SF-36 (BP)	73/67	0.071 (0.169)	4
						VAS (knee)	73/67	0.216 (0.170)	2
				Disability		WOMAC	73/67	0.085 (0.169)	2
						SF-36 (PF)	73/67	0.236 (0.170)	1
Berman et al. 2004	Ann. Intern. Med.	Acupuncture versus sham acupuncture	381	Disability	Unclear	WOMAC	142/141	0.201 (0.146)	1
						SF-36 (PCS)	142/141	0.134 (0.146)	2
		Education versus sham acupuncture	380	Disability	Unclear	WOMAC	141/108	−0.244 (0.154)	2
						SF-36 (PCS)	141/108	−0.255 (0.154)	1
Burch et al. 2008	Osteoarthritis and Cartilage	Pattern stimulation versus control (TENS)	116	Pain	Unclear	WOMAC	52/53	0.698 (0.201)	1
						VAS (global)	52/53	0.231 (0.195)	2
Case et al. 2003	Ann. Intern. Med.	Diclofena versus placebo	53	Pain	Unclear	WOMAC	25/28	0.446 (0.338)	1
						Lequesne	25/28	0.190 (0.334)	2
		Acetaminophen versus placebo	57	Pain	Unclear	WOMAC	29/28	0.096 (0.326)	2
						Lequesne	29/28	0.217 (0.326)	1
Clegg et al. 2006	N. Engl. J. Med.	Glucosamine + chondroitin versus placebo	630	Pain	Inadequate	WOMAC	317/313	0.127 (0.126)	2
						HAQ	317/313	0.146 (0.126)	1
				Disability		WOMAC	317/313	0.137 (0.126)	1
						HAQ	317/313	0.104 (0.126)	2
		Glucosamine versus placebo	630	Pain	Inadequate	WOMAC	317/313	−0.028 (0.126)	1
						HAQ	317/313	−0.021 (0.126)	2
				Disability		WOMAC	317/313	−0.013 (0.126)	2
						HAQ	317/313	0.056 (0.126)	1
		Chondroitin versus placebo	631	Pain	Inadequate	WOMAC	318/313	−0.020 (0.126)	2
						HAQ	318/313	−0.046 (0.126)	1
				Disability		WOMAC	318/313	0.023 (0.126)	2
						HAQ	318/313	0.029 (0.126)	1
		Celecoxib versus placebo	631	Pain	Inadequate	WOMAC	318/313	0.132 (0.126)	1
						HAQ	318/313	0.130 (0.126)	2
				Disability		WOMAC	318/313	0.179 (0.126)	1
						HAQ	318/313	0.114 (0.126)	2
Forestier et al. 2009	Ann. Rheum. Dis.	Spa therapy versus control	451	Pain	Adequate	VAS (knee)	193/186	0.310 (0.103)	1
						SF-36 (BP)	194/185	0.190 (0.103)	2
				Disability		WOMAC	179/172	0.366 (0.108)	1
						SF-36 (PCS)	190/177	0.153 (0.105)	2
						SF-36 (PF)	195/186	0.070 (0.103)	3
Geba et al. 2002	JAMA	Rofecoxib 25 mg/d versus acetaminophen	189	Pain	Inadequate	VAS (walking)	95/94	0.467 (0.208)	1
						VAS (night)	95/94	0.394 (0.207)	3
						VAS (rest)	95/94	0.411 (0.206)	2
		Rofecixib 12.5 mg/d versus acetaminophen	190	Pain	Inadequate	VAS (walking)	96/94	0.191 (0.206)	1
						VAS (night)	96/94	0.069 (0.206)	3
						VAS (rest)	96/94	0.135 (0.206)	2
		Celecoxib versus Acetaminophen	191	Pain	Inadequate	VAS (walking)	97/94	0.233 (0.206)	1
						VAS (night)	97/94	−0.043 (0.206)	3
						VAS (rest)	97/94	0.074 (0.206)	2
Gibofsky et al. 2003	Arthritis, Rheum.	Rofecoxib versus placebo	285	Pain	Unclear	WOMAC	189/96	0.489 (0.163)	1
						VAS (walking)	189/96	0.366 (0.162)	2
		Celecoxib versus placebo	286	Pain	Unclear	WOMAC	190/96	0.514 (0.163)	1
						VAS (walking)	190/96	0.451 (0.163)	2
Hinman et al. 2003	BMJ	Tappening versus placebo	58	Pain	Unclear	WOMAC	29/29	0.160 (0.318)	4
						NRS (activity)	29/29	0.802 (0.329)	1
						NRS (walking)	29/29	0.795 (0.329)	2
						SF-36 (BP)	29/29	0.308 (0.320)	3
				Disability		WOMAC	29/29	1.019 (0.340)	1
						SF-36 (PF)	29/29	0.247 (0.323)	2
		Control tape versus placebo	58	Pain	Unclear	WOMAC	29/29	0.649 (0.329)	1
						NRS (activity)	29/29	0.376 (0.329)	4
						NRS (walking)	29/29	0.649 (0.329)	1
						SF-36 (BP)	29/29	0.431 (0.325)	3
				Disability		WOMAC	29/29	0.640 (0.329)	1
						SF-36 (PF)	29/29	0.230 (0.323)	2
Hughes and Carr 2002	Rheumatology	Glucosamine sulphate versus placebo	80	Pain	Unclear	WOMAC	39/39	−0.066 (0.227)	3
						VAS (activity)	39/39	−0.020 (0.226)	5
						VAS (knee)	39/39	−0.032 (0.226)	4
						VAS (rest)	39/39	0.323 (0.228)	1
						McGill (sense)	39/39	0.221 (0.227)	2
Kirkley et al. 2008	N. Engl. J. Med.	Arthroscopic surgery versus placebo	188	Pain	Adequate	WOMAC	88/80	0.316 (0.155)	1
						ASES	88/80	−0.073 (0.155)	2
				Disability		WOMAC	88/80	0.259 (0.155)	1
						ASES	88/80	0.209 (0.155)	2
						SF-36 (PCS)	88/80	−0.009 (0.155)	3
Kitay et al. 2009	Osteoarthritis and Cartilage	Vibrations, CPM, and heat versus sham device	71	Pain	Adequate	WOMAC	24/32	0.984 (0.284)	1
						VAS (knee)	24/32	0.984 (0.284)	1
Mazières et al. 2007	Ann. Rheum. Dis.	Chondroitin sulphate versus placebo	307	Pain	Unclear	VAS (activity)	139/140	0.260 (0.120)	1
						VAS (rest)	139/140	0.092 (0.120)	2
McCarthy et al. 2004	Rheumatology	Class-based versus home-based exercise	214	Pain	Unclear	WOMAC	111/103	0.321 (0.138)	2
						VAS (walking)	111/103	0.836 (0.143)	1
Moseley et al. 2002	N. Engl. J. Med.	Arthroscopic debridement versus placebo	119	Pain	Unclear	Knee specific			
						Pain scale	53/55	0.010 (0.232)	3
						AIMS (pain)	53/55	−0.072 (0.224)	2
						SF-36 (BP)	52/55	0.114 (0.194)	1
		Arthroscopic lavage versus placebo	121	Pain	Unclear	Knee specific			
						Pain scale	55/55	0.110 (0.236)	2
						AIMS (pain)	56/55	−0.200 (0.223)	1
						SF-36 (BP)	57/55	0.090 (0.189)	3
Neustadt et al. 2005	J. Rheumatol.	Hyalerone four times a week versus control	251	Pain	Unclear	WOMAC	115/114	0.098 (0.162)	2
						VAS (activity)	104/100	0.158 (0.172)	1
		Hyalerone three times a week versus control	242	Pain	Unclear	WOMAC	107/114	−0.028 (0.164)	2
						VAS (activity)	90/100	0.030 (0.176)	1
Niethard et al. 2005	J. Rheumatol.	Diclofena versus placebo	238	Pain	Unclear	WOMAC	117/120	0.363 (0.131)	1
						VAS (activity)	117/119	0.360 (0.131)	2
Nuñez et al. 2006	Osteoarthritis and Cartilage	Education programme versus control	100	Pain	Unclear	WOMAC	43/37	0.980 (0.237)	1
						SF-36 (BP)	43/37	0.605 (0.229)	2
				Disability		WOMAC	43/37	0.742 (0.232)	1
						SF-36 (PF)	43/37	0.576 (0.229)	2
J. Petrella and M. Petrella 2006	Arch. Intern. Med.	Massage versus control	74	Pain	Adequate	WOMAC	34/34	0.948 (0.256)	1
						VAS (knee)	34/34	0.872 (0.254)	2
Petrella et al. 2006	J. Rheumatol.	Hyaleron versuscontrol	106	Pain	Inadequate	WOMAC	53/53	0.092 (0.194)	1
						VAS (activity)	53/53	−0.040 (0.194)	2
						VAS (walking)	53/53	0.000 (0.194)	3
				Disability	Unclear	WOMAC	53/53	−0.147 (0.195)	2
						SF-36 (PF)	53/53	0.084 (0.194)	1
Petrella 2002	Arch. Intern. Med.	Hyalerone + placebo versus control	53	Pain	Unclear	WOMAC	25/28	0.190 (0.384)	4
						VAS (activity)	25/28	0.890 (0.398)	1
						VAS (rest)	25/28	−0.539 (0.389)	3
						VAS (walking)	25/28	0.623 (0.391)	2
		Hyalerone + NSAID versuscontrol	57	Pain	Unclear	WOMAC	29/28	0.238 (0.377)	4
						VAS (activity)	29/28	0.501 (0.381)	2
						VAS (rest)	29/28	0.383 (0.379)	3
		NSAID + saline versus control	54	Pain	Unclear	VAS (walking)	29/28	1.268 (0.403)	1
						WOMAC	26/28	0.336 (0.384)	3
						VAS (activity)	26/28	1.622 (0.428)	1
						VAS (rest)	26/28	0.173 (0.382)	4
						VAS (walking)	26/28	1.154 (0.406)	2
Pham et al. 2004	Ann. Rheum. Dis.	Hyalerone versus control	206	Pain	Unclear	VAS (knee)	131/85	−0.035 (0.177)	2
						Painful days	131/85	−0.078 (0.177)	1
		Diacerein versus control	170	Pain	Unclear	VAS (knee)	85/85	−0.023 (0.188)	2
						Painful days	85/85	−0.027 (0.188)	1
Ravaud et al. 2009	BMJ	Standardised consultation versus usual care	327	Disability	Unclear	WOMAC	146/181	0.260 (0.112)	2
						SF-12 (PCS)	129/147	0.361 (0.122)	1
Raynauld et al. 2003	Arthritis Rheum.	Triamcinolone versus saline (control)	68	Pain	Unclear	WOMAC	33/33	0.307 (0.248)	2
						VAS (knee)	33/33	0.672 (0.253)	1
Raynauld et al. 2002	Osteoarthritis and Cartilage	Hylan G-F 20 versus Control	255	Disability	Unclear	WOMAC	124/107	0.668 (0.136)	1
						SF-36 (PCS)	124/107	0.553 (0.134)	2
Rodrigues et al. 2008	Arthritis Rheum.	Medial-wedge insole versus neutral insole	30	Pain	Adequate	VAS (activity)	16/14	1.337 (0.405)	1
						VAS (rest)	16/14	0.899 (0.384)	2
						VAS (night)	16/14	0.802 (0.380)	3
Rooks et al. 2006	Arthritis Rheum	Preoperative exercise versus control	45	Pain	Unclear	WOMAC	14/15	0.220 (0.373)	2
						SF-36 (BP)	14/15	−0.322 (0.374)	1
				Disability		WOMAC	14/15	0.034 (0.372)	2
						SF-36 (PF)	14/15	−0.391 (0.375)	1
Sengupta et al. 2008	Arthritis Res. Ther.	5-Loxin 250 mg versus control	50	Pain	Unclear	WOMAC	23/23	1.292 (0.393)	2
						VAS (knee)	23/23	2.463 (0.467)	1
		5-Loxin 100 mg versus control	50	Pain	Unclear	WOMAC	24/23	1.039 (0.379)	1
						VAS (knee)	24/23	1.918 (0.425)	2
Song et al. 2009	Ann. Rheum. Dis.	Icatibant 2000 *μ*g versus control	29	Pain	Unclear	VAS (activity)	14/15	0.492 (0.459)	1
						VAS (rest)	14/15	0.391 (0.456)	2
		Icatibant 500 *μ*g versus control	27	Pain	Unclear	VAS (activity)	12/15	0.411 (0.470)	1
						VAS (rest)	12/15	0.391 (0.470)	2
Tannenbaum et al. 2004	Ann. Rheum. Dis.	Lumiracoxib 400 mg versus placebo	734	Pain	Unclear	WOMAC	491/243	0.211 (0.132)	2
						VAS (knee)	491/243	0.307 (0.132)	1
		Lumiracoxib 200 mg versus placebo	730	Pain	Unclear	WOMAC	487/243	0.189 (0.132)	2
						VAS (knee)	487/243	0.236 (0.132)	1
		Celecoxib versus placebo	724	Pain	Unclear	WOMAC	481/243	0.184 (0.132)	2
						VAS (knee)	481/243	0.217 (0.132)	1
Trnavský et al. 2004	J. Rheumatol.	Ibuprofen versus placebo	50	Pain	Unclear	VAS (walking)	25/25	1.739 (0.332)	1
						VAS (rest)	25/25	0.932 (0.298)	2
						VAS (knee)	25/25	0.786 (0.294)	3
Vas et al. 2004	BMJ	Acupuncture versus control	97	Pain	Unclear	WOMAC	48/49	1.109 (0.218)	2
						VAS (knee)	48/49	1.249 (0.222)	1
Wang et al. 2009	Arthritis Rheum.	Tai chi versus control	40	Pain	Adequate	WOMAC	20/20	0.456 (0.320)	1
						VAS (knee)	20/20	0.015 (0.316)	2
			40	Disability	Adequate	WOMAC	20/20	0.356 (0.319)	2
						SF-36 (PCS)	20/20	0.820 (0.329)	1
Witt et al. 2005	Lancet	Acupuncture versus minimal acupuncture	226	Pain	Unclear	WOMAC	149/75	0.150 (0.142)	1
						SES	149/75	0.062 (0.142)	2
			226	Disability	Unclear	WOMAC	149/75	0.249 (0.142)	1
						SF-36 (PCS)	149/75	0.215 (0.142)	3
						PDI	149/75	0.245 (0.142)	2
Wittenberg et al. 2006	Arthritis Rheum.	Lumiracoxib versus placebo	219	Pain	Unclear	WOMAC	144/75	0.406 (0.185)	2
						VAS (activity)	144/75	0.645 (0.186)	1
		Celecoxib versus placebo	220	Pain	Unclear	WOMAC	145/75	0.396 (0.184)	2
						VAS (activity)	145/75	0.525 (0.185)	1

WOMAC (Western Ontario and McMaster Universities Arthritis Index), VAS (visual analogue scale), NRS (numerical rating scale), AIMS (Arthritis Impact Measurement Scale), ASES (Arthritis Self-Efficacy Scale), HAQ (Health Assessment Questionnaire), PDI (Pain Disability Index), SES (Schmerzempfindungsskala) (Pain Experience Scale), SF-36 (Short Form 36), BP (bodily pain), PF (physical function), PCS (Physical Composite Scale).

**Table 2:** Frequency of the used outcomes listed according to their frequency in the included trials.

Pain outcome (scale)	Number of trials *k* = 35 (%)	Number of substudies *k* = 54 (%)	Mean rank in responsiveness (range)
WOMAC pain (Likert scale)	15 (43%)	20 (37%)	1.8 (1–4)
Global knee pain (VAS)	13 (37%)	17 (31%)	1.7 (1–4)
WOMAC pain (100 mm scale)	12 (34%)	21 (39%)	1.9 (1–4)
Pain during activity (VAS)	12 (34%)	18 (33%)	1.4 (1–5)
Pain during walking (VAS)	7 (20%)	12 (22%)	1.5 (1–3)
Pain at rest (VAS)	7 (20%)	12 (22%)	2.3 (1–4)
SF-36 (bodily pain (BP) subscale)	6 (17%)	8 (15%)	2.3 (1–4)
Pain at night (VAS)	2 (6%)	4 (7%)	3.0 (3)
HAQ (pain subscale)	1 (3%)	4 (7%)	1.5 (1-2)
Lequesne algofunctional index (pain subscale)	1 (3%)	2 (4%)	1.5 (1-2)
Pain during activity (NRS)	1 (3%)	2 (4%)	2.5 (1–4)
Pain during walking (NRS)	1 (3%)	2 (4%)	2.5 (2-3)
Number of painful days (days)	1 (3%)	2 (4%)	1.0 (1)
AIMS (pain subscale)	1 (3%)	2 (4%)	1.5 (1-2)
Knee-Specific Pain Scale (KSPS)	1 (3%)	2 (4%)	2.5 (2-3)
McGill Pain Questionnaire (pain intensity)	1 (3%)	1 (2%)	2.0 (2)
ASES (pain subscale)	1 (3%)	1 (2%)	2.0 (2)
SES (Schmerzempfindungsskala)	1 (3%)	1 (2%)	2.0 (2)

Disability outcome (scale)	Number of trials *k* = 15 (%)	Number of substudies *k* = 21 (%)	Mean rank in responsiveness (range)

Physical composite score (PFC) (based on SF-36, SF-12, or SF-8)	9 (60%)	11 (52%)	1.8 (1–3)
WOMAC function (100 mm scale)	8 (53%)	13 (62%)	1.5 (1-2)
WOMAC function (Likert scale)	7 (47%)	8 (38%)	1.5 (1-2)
SF-36 (physical function PF subscale)	7 (47%)	8 (38%)	1.8 (1-2)
HAQ (disability subscale)	1 (7%)	4 (19%)	1.5 (1-2)
PDI (pain disability index)	1 (7%)	1 (5%)	2.0 (2)
ASES (disability subscale)	1 (7%)	1 (5%)	2.0 (2)

WOMAC (Western Ontario and McMaster Universities Arthritis Index), VAS (visual analogue scale), NRS (Numerical Rating Scale), AIMS (Arthritis Impact Measurement Scale), ASES (Arthritis Self Efficacy Scale), HAQ (Health Assessment Questionnaire), PDI (Pain Disability Index), SES (Schmerzempfindungsskala) (Pain Experience Scale), SF-36 (Short Form 36), SF-12 (Short Form 12), SF-8 (Short Form 8).

**Table 3:** A prioritized list of patient-reported outcomes for extracting data in meta-analysis.

*Pain*	
outcome (scale)	
(1) WOMAC pain subscale (Likert/100 mm)	
(2) Pain during activity (VAS)	
(3) Pain during walking (VAS)	
(4) Global knee pain (VAS)	
(5) Pain at rest (VAS)	
(6) SF-36 (bodily pain (BP) subscale)	
(7) HAQ (pain subscale), Lequesne algofunctional index (pain subscale), AIMS (pain subscale), Knee-Specific Pain Scale (KSPS), McGill Pain Questionnaire (pain intensity), ASES (pain subscale), SES (Schmerzempfindungsskala)	
(8) Pain at night (VAS), pain during activity (NRS), pain on walking (NRS), number of painful days (days)	

*Disability*	
outcome (scale)	

(1) WOMAC subscale function (Likert/100 mm)	
(2) SF-36 (subscale physical function (PF))	
(3) Physical composite score (PCS) based on SF-36, SF-12, or SF-8	
(4) HAQ (disability subscale), PDI (pain disability index), ASES (disability subscale)	

WOMAC (Western Ontario and McMaster Universities Arthritis Index), VAS (visual analogue scale), NRS (Numerical Rating Scale), AIMS (Arthritis impact measurement scale), ASES (Arthritis Self Efficacy Scale), HAQ (health assessment questionnaire), PDI (Pain Disability Index), SES (Schmerzempfindungsskala) (Pain Experience Scale), SF-36 (Short Form 36), SF-12 (Short Form 12), SF-8 (Short Form 8).
